# Unicentric Castleman's Disease of Paraspinal Origin Requiring Differentiation From Schwannoma: Case Report

**DOI:** 10.1002/rcr2.70099

**Published:** 2025-01-23

**Authors:** Hazuki Fujimoto, Kayoko Okamura, Shunsuke Tauchi, Rei Tsukamoto, Nanami Kida, Miho Ikeda, Yukihisa Hatakeyama, Hisashi Ohnishi

**Affiliations:** ^1^ Department of Respiratory Medicine Akashi Medical Center Akashi Japan; ^2^ Department of Thoracic Surgery Akashi Medical Center Akashi Japan

**Keywords:** paravertebral region, schwannoma, unicentric Castleman's disease

## Abstract

Unicentric Castleman's disease (UCD) typically presents as an asymptomatic tumour in the anterior or middle mediastinum. Occurrence in the paravertebral region is comparatively rare and it requires differentiation from neurogenic tumours by imaging. In our patient, preoperative imaging findings were atypical of schwannoma. Contrast‐enhanced MRI of the thoracic region showed a muscle‐like mass in contact with the pleura on T1‐weighted images. T2‐weighted images showed high signal, especially at the margins. Diffusion‐weighted images showed diffusion restriction around the limbus, and contrast‐enhanced T1‐weighted images displayed strong enhancement of the mass. Diagnosis could not be made preoperatively, although UCD was suspected. Thoracoscopic tumour resection was performed for definitive diagnosis and treatment and postoperative diagnosis was hyaline‐vascular‐type UCD. Diagnosis of neurogenic tumours can be difficult by imaging alone. When imaging findings are atypical, a definitive diagnosis is required, with consideration of the possibility of UCD.

## Graphical abstract

Unicentric Castleman's disease is often found as an asymptomatic tumour, usually in the chest, specifically the anterior or middle mediastinum. We report a case of unicentric Castleman's disease in the paravertebral region that was diagnosed after surgery. Neurogenic tumours can be difficult to diagnose using imaging alone. When imaging findings are atypical, a definitive diagnosis should be made, with consideration of the possibility of UCD.

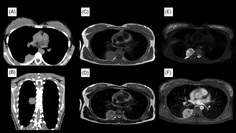

## 1 Introduction

Castleman's disease (CD) is a lymph node hyperplasia with an uncertain aetiology. It is clinically classified as either unicentric (UCD) or multicentric types. UCD has a high frequency of occurrence in the anterior and middle mediastinum, and there are rare reports of paravertebral involvement [1]. We report the case of a patient with UCD in the paravertebral region that notably required careful differentiation from schwannoma.

## 2 Case Report

**FIGURE 1:**
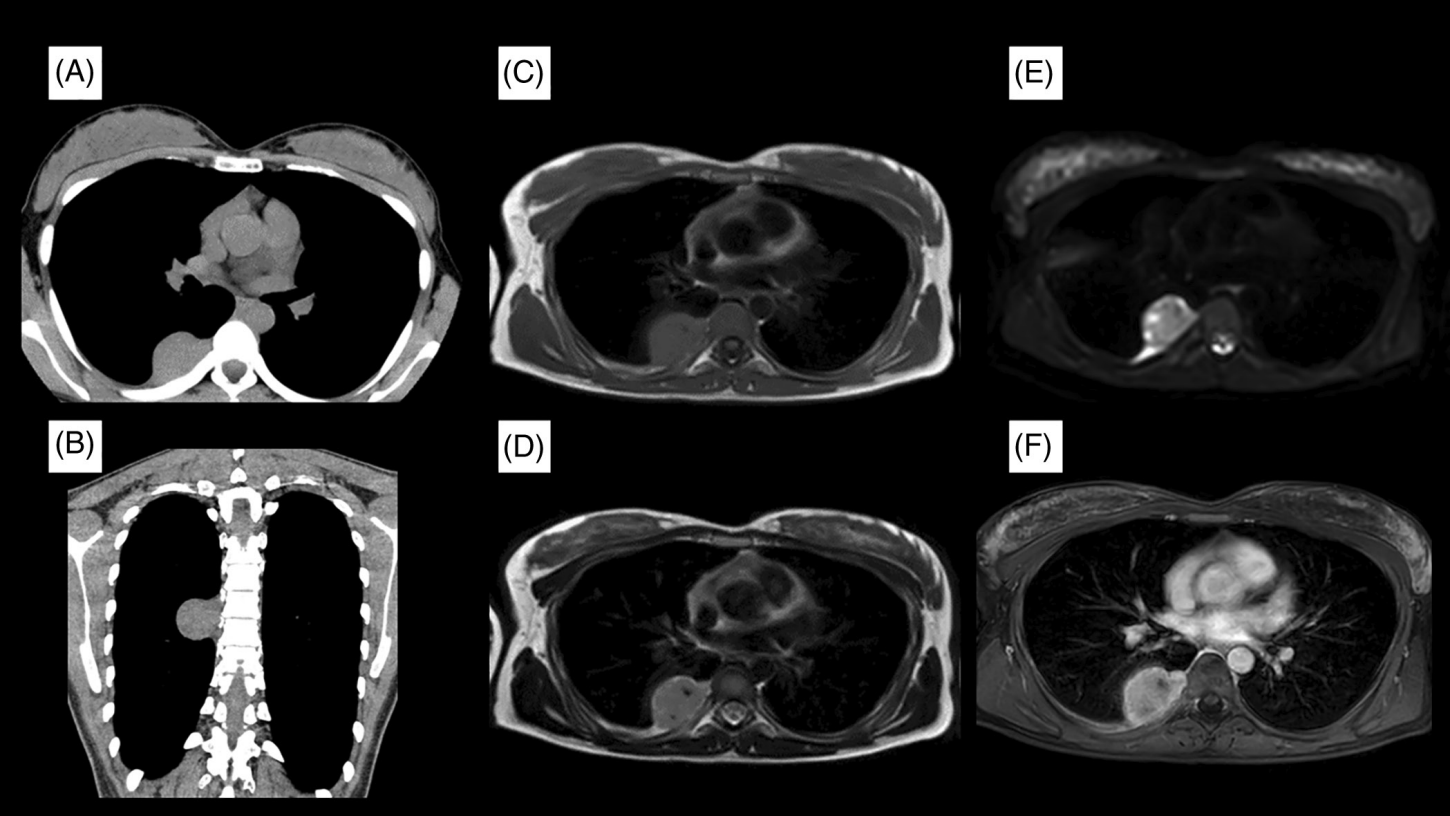
(A and B) Chest CT (mediastinal window) shows a uniform, well‐defined mass measuring approximately 40 mm in length near the vertebral body of the seventh thoracic vertebra. (C) Chest MRI (T1WI) shows a mass adjacent to the pleura with signal intensity comparable to muscle. (D) Chest MRI (T2WI) shows the edges were high‐signal. (E) Chest MRI (diffusion weighted images) shows diffusion restriction around the limbus of the mass. (F) Chest MRI (Contrast‐enhanced T1WI) shows strong, homogeneous enhancement of the mass.

**FIGURE 2:**
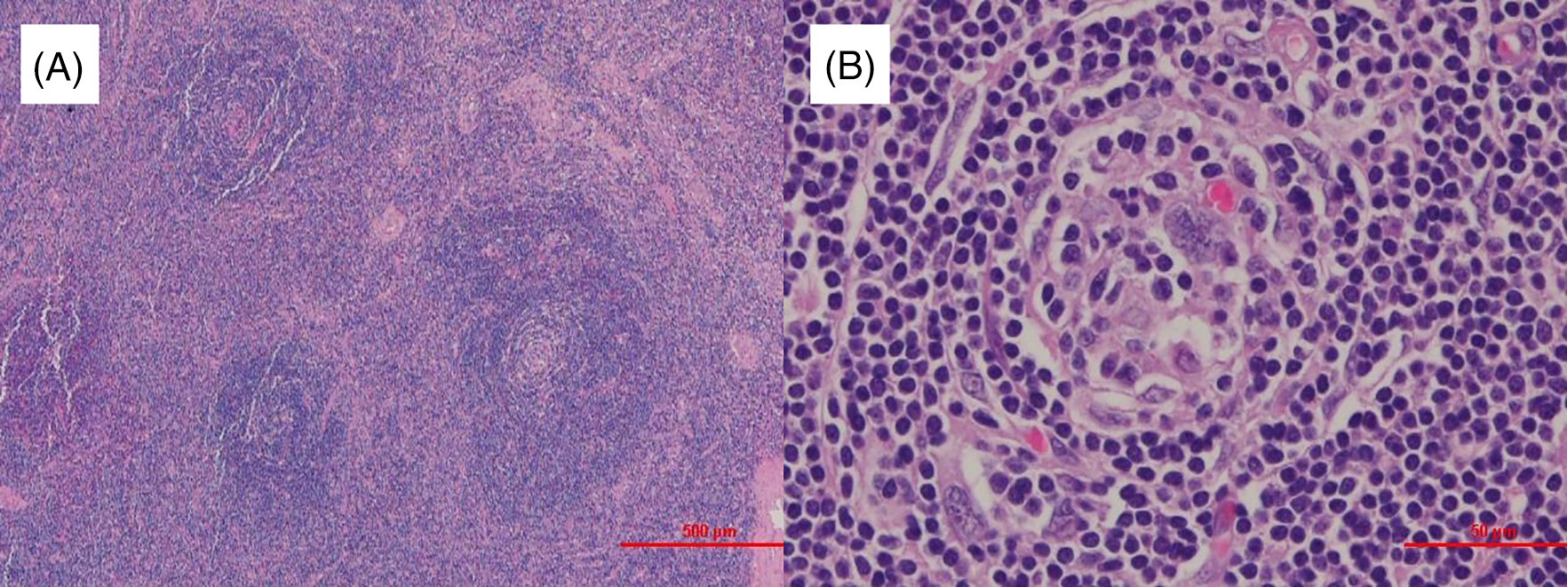
(A) Histopathology examination of the surgical specimen (haematoxylin and eosin stain) shows a marked increase in the vascular components between lymphoid follicles; magnification: ×40. (B) Lymph follicular formation is inconspicuous, with scattered onion‐skin structures reflecting mantle layer development; magnification: ×200.

A Japanese woman in her late twenties, a non‐smoker without previous medical or family history, was referred to our hospital for a close examination after routine chest radiography detected a right hilar mass. She was asymptomatic, and vital signs were normal (blood pressure was 101/69 mmHg, pulse 103/min, respiratory rate of 16/min with an O_2_ saturation of 98% on room air). Her respiratory sounds were clear, the heartbeat was normal, and there were no other remarkable findings. Blood tests revealed no abnormalities. Chest radiography confirmed the mass in the right pulmonary hilar region, and thoracic computed tomography (CT) showed it was a uniform, uncalcified mass of about 40 mm in length near the vertebral body of the seventh thoracic vertebra. The mass was in contact with the pleura and thoracic vertebrae but it did not extend into the intervertebral foramen (Figure 1A,B). T1‐weighted images (T1WI) showed a mass with muscle‐like consistency that was in contact with the pleura (Figure 1C). T2‐weighted images (T2WI) showed that it had high signal, especially at the margins (Figure 1D). Diffusion‐weighted images showed diffusion restriction around the limbus (Figure 1E), and contrast‐enhanced T1WI showed strong contrast of the mass (Figure 1F). CT findings suggested that the mass was schwannoma, but the T1WI and T2WI findings were atypical for schwannoma. Ultrasound‐guided needle biopsy was therefore performed for differential diagnosis. The histopathological findings were mixed T‐ and B‐cell lymphoid aggregates with little atypia and some vitreous‐like deposits in the vessel walls. Based on these results, we suspected UCD and performed thoracoscopic tumour resection for definitive diagnosis and treatment. Intraoperatively, we confirmed a raised tumour lesion near the seventh thoracic vertebra with angiogenesis. The tumour and surrounding connective tissue were excised en bloc. Histopathology of the surgical specimen showed an island‐like vitrified fibrous component within the reactive enlarged lymphoid tissue. Lymph follicle formation was inconspicuous, with scattered onion‐skin structures due to the development of the mantle layer and a marked increase in the vascular component between lymph follicles. There was no atypia in the small lymphocytes, and no increase in plasma cells (Figure 2). Based on the histological findings, we postoperatively diagnosed hyaline‐vascular type UCD. The postoperative course was uneventful, and 2 years after resection the patient remains disease‐free without recurrence.

## 3 Discussion

CD is a lymph node hyperplasia of uncertain aetiology. Clinically, it can be classified as either unicentric or multicentric, and histologically it can be classified as either the asymptomatic hyaline‐vascular type or the plasma cell type, which may cause systemic symptoms. Diagnosis is confirmed through pathological evaluation. The hyaline‐vascular type is unicentric in the majority of cases and usually presents as an asymptomatic mass lesion with a benign course [2]. UCD is expected to be cured by surgical resection. Conversely, most plasma cell type lesions are multicentric, and anti‐inflammatory therapy is often used to treat systemic symptoms.

UCD most frequently occurs in the thorax, accounting for 29% of 235 reported surgical cases [2]. The disease is relatively common in the anterior and middle mediastinum, where lymphoid tissue is abundant, but it is rare in the paravertebral region, with just 19 confirmed cases (Table 1). Hyaline‐vascular type CD typically appears on CT as a well‐defined mass with uniform density, isoattenuating with muscle. Contrast enhanced CT is strongly and homogeneously enhanced due to the presence of abundant blood flow. T1WI MRI shows a slightly higher signal than muscle, and T2WI shows a much higher signal. Diffusion‐weighted images are characterised by marked diffusion restriction, while contrast enhanced images show early and strong contrast [3]. Schwannomas share imaging features with hyaline‐vascular CD, for example the smooth margins and homogeneous internal structure, but they may exhibit a mottled appearance or target signs. This reflects a mixture of Antoni A areas with high cellular component in which there is clear contrast and Antoni B areas with mucous degeneration in which the contrast is unclear. Treatment of UCD is surgical resection, and if total resection is possible, the 10‐year survival rate is 90% [2]. Similarly, surgical resection is the standard treatment for schwannomas [4].

**TABLE 1:** Summary of the 19 reported cases.

Age (years)	Sex	Imaging diagnosis	Total resection	Recurrence
59	Female	Bronchogenic cyst	Not described	No recurrence
40	Male	Neurogenic tumour	Possible	No recurrence
36	Female	Not described	Possible	No recurrence
52	Male	Not described	Possible	No recurrence
18	Male	Solitary fibrous tumour	Possible	No recurrence
44	Male	Not described	Possible	No recurrence
19	Female	Neurogenic tumour	Intraoperatively, she was diagnosed with lymphoma and surgery was aborted. The final diagnosis was CD. Postoperative radiation therapy was performed.	No recurrence
31	Female	Schwannoma, neurofibroma, paraganglioma, sarcoidosis, lymphoma	Not described	No recurrence
30	Female	Not described	Possible	No recurrence
50	Male	Not described	Not described	No recurrence
60	Female	Lung cancer	Possible	No recurrence
41	Male	Neurogenic tumour, lipoma	Possible	No recurrence
53	Male	Neurogenic tumour	Possible	No recurrence
28	Female	Not described	Possible	No recurrence
45	Male	Neurogenic tumour, solitary fibrous tumour	Not described	No recurrence
21	Female	Not described	Not described	No recurrence
19	Male	Neurogenic tumour	Possible	No recurrence
19	Female	Lung cancer	Possible	No recurrence
34	Male	Not described	Not described	No recurrence

In the present case, we suspected schwannoma, but ultimately UCD was diagnosed because the contrast‐enhanced MRI of the tumour was strongly homogeneous, a finding that is not typical of schwannoma. UCD occurring in the paravertebral region, as in this case, is rare. The differential disease for mediastinal tumours arising in the paravertebral region is neurogenic tumours, including schwannomas [5]. Of the 19 reported cases, seven were suspected to be neurogenic tumours on imaging, as in the present case, and none were suspected to have been CD. In all cases, the diagnosis of UCD was surgically confirmed, and there were no cases of postoperative recurrence.

Neurogenic tumours can be difficult to diagnose using imaging alone. When imaging findings are atypical for neurogenic tumours, a definitive diagnosis should be made, with consideration of the possibility of UCD, although this is a rare condition. Treatment is nonetheless by resection.

## Author Contributions

All authors contributed to the conception of the case report. Hazuki Fujimoto wrote and drafted the manuscript. All authors have read and approved the final version of the manuscript.

## Ethics Statement

The authors declare that appropriate written informed consent was obtained for the publication of this manuscript and accompanying images.

## Conflicts of Interest

The authors declare no conflicts of interest.

## Data Availability

Data sharing is not applicable to this article as no new data were created or analyzed in this study.
